# Clinical and pathophysiological links between hypoglycemia and cardiovascular risk in type 2 diabetes mellitus

**DOI:** 10.3389/fcdhc.2026.1765597

**Published:** 2026-02-05

**Authors:** Lamija Ferhatbegović, Minela Bećirović, Emir Bećirović, Sumeja Sarajlić, Aida Ribić, Asja Šarić, Amir Bećirović, Belma Pojskić

**Affiliations:** 1 Department of General Internal Medicine, Cantonal Hospital Zenica, Zenica, Bosnia and Herzegovina; 2 Medical Faculty, University of Zenica, Zenica, Bosnia and Herzegovina; 3 Clinic for Internal Diseases, University Clinical Center Tuzla, Tuzla, Bosnia and Herzegovina; 4 Department of Pediatrics, General Hospital “Prim.dr. Abdulah Nakaš”, Sarajevo, Bosnia and Herzegovina

**Keywords:** autonomic dysfunction, cardiovascular diseases, endothelial dysfunction, hypoglycemia, type 2 diabetes

## Abstract

Severe hypoglycemia increases the risk of cardiovascular disease (CVD) in people with diabetes. Large cohort studies and scientific statements show that severe hypoglycemia is linked to higher rates of coronary heart disease, cardiovascular events, and mortality in both type 1 and type 2 diabetes. This risk is especially high in individuals with significant vascular risk, such as older adults and those with multiple cardiovascular risk factors. Hypoglycemia triggers several pathophysiological changes that increase cardiovascular risk. These include activation of the sympathoadrenal system, promotion of proinflammatory and prothrombotic states, arrhythmogenic changes, and increased hemodynamic stress. Experimental evidence shows that recurrent hypoglycemia worsens microvascular dysfunction and promotes adverse cardiac remodeling, especially in people with diabetes. While the link between hypoglycemia and cardiovascular events is well established, the causality remains debated. Hypoglycemia may directly contribute to cardiovascular disease or indicate underlying vulnerability, especially in patients with advanced disease or comorbidities. Minimizing hypoglycemic episodes is recommended for all patients with diabetes, particularly those with established cardiovascular disease, due to the clear association with adverse outcomes.

## 1 Introduction

Atherosclerotic cardiovascular diseases (ASCVD) remain the main cause of morbidity and mortality in diabetes (1). Despite the significant advancements in diabetes management over last decades and consequently reduced complication rates, diabetes is attributed for 10.7% of all global deaths among individuals aged 20–79 years, with approximately half of these deaths resulting from cardiovascular diseases (2, 3). The landmark UK Prospective Diabetes Study (UKPDS) demonstrated that intensive glycemic control in patients with newly diagnosed diabetes over extended period of time prevents microvascular complications (4). However, this traditional concept has been challenged by findings from more recent large scale clinical trials, which not only failed to demonstrate cardiovascular benefit from intensive glycemic treatment, but also revealed negative effects, mainly attributed to hypoglycemia (5–7).

Hypoglycemia, particularly in its severe form, has been consistently associated with an increased risk of cardiovascular events and mortality, however whether this relationship reflects causality or identifies vulnerable high-risk patients remains controversial (8–10). Severe hypoglycemia represents a major challenge in diabetes management, especially among patients treated with insulin or insulin secretagogues. Importantly, accumulating evidence suggest that hypoglycemia may function more as a marker of cardiovascular vulnerability rather than a direct causal factor. Hypoglycemia is defined as a blood glucose concentration below 70 mg/dL (<3.9 mmol/L) (11–16). According to the American Diabetes Association and other expert consensus groups, hypoglycemia is categorized into three levels. Level 1 (glucose <70 mg/dL [<3.9 mmol/L] and ≥54 mg/dL [≥3.0 mmol/L]) marks the threshold at which counter-regulatory hormonal responses, such as adrenergic symptoms (shakiness, tachycardia, perspiration, and hunger), are typically triggered in non-diabetic individuals. In patients with diabetes, however, impaired awareness and blunted counter-regulatory responses may result in asymptomatic hypoglycemia (11, 14). Level 2 (glucose <54 mg/dL [<3.0 mmol/L]) is clinically significant, as neuroglycopenic manifestations, such as confusion, irritability, slurred speech, or altered consciousness, can occur, necessitating prompt corrective action to prevent neurological injury (11, 13). Level 3 (severe hypoglycemia) is characterized by marked cognitive or physical dysfunction requiring external assistance for recovery, regardless of the measured glucose level (11, 13). Common precipitating factors for hypoglycemia include excessive doses of exogenous insulin, sulfonylurea therapy, other antihyperglycemic agents, prolonged fasting, metabolic disturbances, and underlying hormonal deficiencies (12, 17, 18). Recurrent hypoglycemia in patients with diabetes can significantly impair quality of life, reduce treatment adherence, and increase the risk of hypoglycemia unawareness, cardiovascular complications, and overall mortality (14, 19). Therefore, severe hypoglycemia requires immediate recognition and intervention to prevent serious adverse outcomes (11, 12, 17).

This review aims to provide a comprehensive understanding of mechanisms by which hypoglycemia acts as an enhancing risk factor for cardiovascular outcomes, as well as its clinical implications and strategies for prevention.

## 2 Pathophysiological mechanisms linking hypoglycemia and cardiovascular events

### 2.1 Autonomic and hemodynamic effects

Repeated episodes of hypoglycemia can diminish the typical autonomic sympathoadrenal responses to decreasing glucose levels, leading to reduced awareness of hypoglycemia and impaired glucose counter-regulation, a condition known as hypoglycemia-associated autonomic failure (HAAF) (13, 18, 19). In HAAF, recurrent hypoglycemia inhibits the release of counter-regulatory hormones, particularly epinephrine, and reduces the manifestation of autonomic symptoms, consequently increasing the risk of subsequent severe hypoglycemia (18, 19). Importantly, unlike diabetic autonomic neuropathy (DAN), HAAF represents a predominantly functional and potentially reversible condition, as restoration of hypoglycemia avoidance can partially or fully normalize autonomic responses (20). In contrast, DAN, and particularly cardiovascular autonomic neuropathy (CAN), reflects a chronic structural and functional impairment of the autonomic nervous system. Autonomic dysfunction, especially CAN, is both a result and a factor that increases the risk of hypoglycemia. The pathophysiology of diabetic neuropathy is complex and multifactorial, but data suggest hyperglycemia as primary mechanism, further involving insulin resistance, dyslipidemia, chronic inflammation, oxidative stress, and mitochondrial dysfunction, leading to progressive neuronal injury and impaired autonomic signaling (21, 22). Individuals with impaired autonomic function have less heart rate variability and decreased sensitivity to baroreflex during hypoglycemic episodes, indicating decreased vagal and sympathetic control of cardiovascular responses (20, 21). This impairment can cause inadequate physiological responses to hypoglycemia, such as failure to increase heart rate or blood pressure appropriately, further elevating the risk of severe and unrecognized hypoglycemia (20, 21). CAN may potentiate the clinical consequences of hypoglycemia by limiting compensatory cardiovascular responses, while hypoglycemia itself may further aggravate autonomic instability (23–26). Autonomic neuropathy predisposes individuals to hypoglycemia by impairing their ability to mount proper physiological responses, including catecholamine release, and by causing symptoms (13, 22). Thus, while HAAF and CAN are distinct entities, they may coexist and interact: recurrent hypoglycemia promotes functional autonomic failure (HAAF), whereas underlying CAN reduces resilience to hypoglycemia and amplifies its cardiovascular impact (23, 27).

The bidirectional relationship between hypoglycemia and autonomic dysfunction highlights the importance of minimizing hypoglycemic episodes to preserve autonomic function and reduce cardiovascular vulnerability.

### 2.2 Electrophysiological and proarrhythmic changes

The first observational evidence suggesting an association between hypoglycemia and sudden death in type 1 diabetes was published in 1991 by Tattersall and Gill (28). Subsequent analysis indicated that hypoglycemia or autonomic dysfunction could be responsible for sudden death and may account for 5-6% of all deaths in type 1 diabetes, a phenomenon commonly referred to as “death in bed syndrome” (29). Experimental studies in both animals and humans have demonstrated that hypoglycemia affects cardiac repolarization leading to proarrhythmic events. A consistently observed alteration is the prolongation of the QT interval corrected by heart rate (QTc), which is recognized risk factor for malignant ventricular arrhythmias (30–34). QTc prolongation has been documented during hypoglycemic episodes in individuals with and without diabetes, and this effect can persist for up to 60 minutes after recovery, regardless of the glucose levels. In one study, nearly two thirds of patients experienced QTc prolongation exceeding 500 ms during hypoglycemia, surpassing the threshold for malignant arrhythmia (31, 32, 35).

Arrhythmogenic risk is further exacerbated by alterations in T-wave symmetry and an elevated Tpeak-to-Tend ratio (32). Additionally, increased ventricular ectopy has been reported during hypoglycemia, which is also associated with sudden cardiac death. In rare cases, low blood glucose can induce atrial fibrillation or even high degree heart block (33, 36).

The frequency of observed electrophysiological changes does not differ between daytime or nighttime episodes. However, nocturnal hypoglycemia, which typically lasts longer than daytime episodes, is associated with a higher incidence of bradycardia, and lower frequency of atrial ectopic beats (30).

The underlying mechanisms are multifactorial. During hypoglycemia, elevated sympathetic activity and catecholamine release can lengthen cardiac repolarization, thereby heightening arrhythmic vulnerability (31–33). Additional influence on cardiac electrophysiology and a contributor to overall arrhythmic risk could be hypokalemia, a frequent consequence of insulin induced hypoglycemia (30, 33). Cardiac autonomic regulation is time-dependent: initial vagal withdrawal increases heart rate, but with sustained hypoglycemia, vagal reactivation can result in bradycardia, especially in individuals with diabetes (32, 33).

The electrophysiological effects of hypoglycemia are evident not only during the acute episode but also persist following recovery, regardless of the achieved glucose level (35, 37). These findings provide a mechanistic foundation for the documented association between hypoglycemia and sudden cardiac death in diabetes, including the “dead-in-bed” syndrome (33).

### 2.3 Endothelial dysfunction and inflammation

Macrovascular and microvascular complications are widespread among individuals with diabetes and are associated with substantial morbidity and mortality (38, 39). The onset of chronic vascular complications and initiation of atherosclerosis in diabetes is rooted in endothelial dysfunction. Oxidative stress, defined as an imbalance in reactive oxygen species (ROS), induces damage to endothelial cells and their intercellular junctions, resulting in increased endothelial permeability and consequently promoting endothelial dysfunction (40). Endothelial dysfunction reflects a transition from a protective endothelial phenotype to one promoting vasoconstriction, inflammation, and thrombosis. It is primarily driven by reduced nitric oxide (NO) bioavailability, oxidative stress, and inflammatory activation, together with an imbalance of vasodilators and vasoconstrictors (41, 42).

Multiple factors, including elevated glucose levels, increased systolic blood pressure, and impaired lipid metabolism, contribute to the endothelial dysfunction in patients with diabetes (38, 43). In the traditional view, hyperglycemia has been considered the predominant driver of endothelial dysfunction in diabetes; however, emerging evidence indicates that hypoglycemia likewise plays a meaningful role in impairing endothelial function (44–47). Studies examining insulin-induced hypoglycemia in both healthy subjects and individuals with diabetes demonstrated that hypoglycemia provokes an inflammatory response through activation of pro-inflammatory mediators (IL-6, IL-8, TNF-α), increases P-selectin–mediated platelet activation, and elevates plasminogen activator inhibitor-1 (PAI-1) levels and the levels of adhesion molecules (ICAM-1,VCAM-1), thereby reducing systemic fibrinolytic capacity and promoting proatherosclerotic processes (48–51). It is noteworthy that the vascular effects of hypoglycemia appear to be prolonged. In the study by Wright et al., these alterations were detectable up to 24 hours after insulin-induced hypoglycemia in individuals with diabetes, with endothelial changes occurring earlier and lasting longer than in healthy subjects (49). Additionally, evidence indicates that alterations in oxidative stress and pyroptosis are more pronounced during recurrent hypoglycemia compared with single acute hypoglycemic episodes (52).

### 2.4 Recurrent hypoglycemia

Repeated hypoglycemia is common in patients with diabetes (53). After several episodes of hypoglycemia the counterregulatory mechanisms are blunted, the awareness of hypoglycemia is impaired and therefore the duration of hypoglycemic episode is prolonged (54). Frequent and prolonged hypoglycemic events maintain a chronic milieu of oxidative stress, inflammatory signaling, and thrombogenic activation, progressively amplifying vascular risk through cumulative exposure (55). Experimental studies consistently demonstrate that recurrent hypoglycemia leads to cognitive impairment, structural brain changes, and even increased mortality (56, 57). In insulin-treated rats, one hour of recurrent hypoglycemia resulted in increased clot weight that persisted for at least seven days post-exposure, compared with only a one-day effect following a single hypoglycemic episode. Accordingly, recurrent hypoglycemia was associated with a heightened risk of thrombosis in these animals (58). Similar effects have been reported in humans, where prior hypoglycemia in individuals with type 2 diabetes induces both immediate and long-lasting thrombotic responses lasting up to seven days, reflected by prolonged clot lysis time, increased clot maximum absorbance, and elevated levels of fibrinogen and complement C3. In contrast, these hypoglycemia-induced changes in healthy subjects tend to resolve rapidly with metabolic recovery (59). The effects of recurrent hypoglycemia may also manifest as subtle impairments in cardiac function. During hypoglycemia, ejection fraction typically increases as a compensatory response; however, the corresponding change in left ventricular global longitudinal strain (GLS) is diminished in individuals with diabetes compared with healthy controls (60). However in large clinical trial there were no association between recurrent hypoglycemia and vascular outcomes or mortality. Notably, the incidence of recurrent hypoglycemia was low, which raises questions regarding the real-world impact of recurrent hypoglycemia (61).

## 3 Clinical evidence on the association between hypoglycemia and cardiovascular outcomes

The landmark UKPDS trial, published in 1998, showed that strict glycemic control in newly diagnosed diabetes patients lowered the risk of microvascular complications and suggested a possible benefit for macrovascular outcomes as well (62). However, later large-scale clinical trials in patients with longer-standing diabetes had challenged this traditional view and further underscored the detrimental impact of hypoglycemia (**Table 1**). Action to Control Cardiovascular Risk in Diabetes (ACCORD) trial was terminated prematurely because the results revealed unexpected detrimental effects of intensive glycemic control, which were considered to be related to hypoglycemia. In this study, patients with long-standing type 2 diabetes were randomized to either intensive glycemic control (target HbA1c <6.0%) or standard therapy. Throughout the follow-up period, the intensive treatment group experienced a higher incidence of severe hypoglycemic episodes, which was accompanied by a 20% increase in mortality compared with the standard therapy group. Owing to these findings, the intensive treatment arm was ended prematurely after a mean follow-up of 3.5 years, rather than the originally planned 5 years (5). Nevertheless, subsequent analyses of the ACCORD trial showed that independent predictors of death were baseline higher HbA1c (> 8.5%), self-reported neuropathy and history of taking aspirin which could reflect higher burdens of microvascular complications and overall risk. When hypoglycemia was more closely analyzed, severe hypoglycemia was identified as definite cause of death in only one case in intensive treatment arm, while as possible/probable cause was not common in either group. The risk of death in intensive group was higher in patients with higher baseline HbA1c and smaller reductions in HbA1c following randomization. In this same subgroup of patients the risk of severe hypoglycemia was higher. In contrast, minor hypoglycemic episodes which occurred more frequently in the intensive treatment arm, were paradoxically associated with lower subsequent mortality. A possible mechanism for this observation could be “hypoglycemic preconditioning,” which protects against brain injury during severe hypoglycemia while also blunting catecholamine release, thereby offering protection from malignant arrhythmias (64, 65).

**Table 1:** Major randomized clinical trials evaluating hypoglycemia and cardiovascular outcomes in type 2 diabetes.

Trial	Population	Intervention	Hypoglycemia findings	Cardiovascular outcomes	Comments
ACCORD (5)	10,251 patients with long-standing T2D, high CV risk	Intensive glycemic control (target HbA1c <6.0%) vs. standard therapy (target HbA1c 7.0-7.9%)	Severe hypoglycemia more frequent in intensive group	Increased all-cause and CV mortality in intensive group	Intensive arm terminated early (mean follow-up 3.5 years), *post hoc* analyses suggest hypoglycemia as a marker of vulnerability rather than a proven causal factor
ADVANCE (6, 61)	11,140 patients with T2D, mean duration 8 years	Intensive glucose lowering (HbA1c ≤6.5%) vs. standard	Severe hypoglycemia associated with higher risk of major CV events and non-cardiovascular outcomes	Higher risk of MI and stroke with severe hypoglycemia	Severe hypoglycemia predicted both CV and non-CV outcomes (respiratory, gastrointestinal, dermatological), supporting its role as a marker of high-risk or frail patients rather than a CV-specific causal factor
VADT (7)	1,791 veterans with poorly controlled long-standing T2D	Intensive vs. standard glycemic control	Recurrent hypoglycemia more common in intensive arm	Increased CV events and mortality in patients experiencing recurrent hypoglycemia	Particularly relevant in patients with high baseline CV risk, long-duration diabetes and older adults
ORIGIN (63)	12,537 patients with early T2D or impaired fasting glucose	Basal insulin vs. standard care	Hypoglycemia observed but lower frequency than ACCORD/ADVANCE	No increase in primary CV outcomes, slight increase in CV risk in patients with recurrent hypoglycemia	Early diabetes population; hypoglycemia less frequent, events identify individuals with increased CV vulnerability

In other large scale clinical trials similar detrimental effects in intensive treatment groups were not observed. A *post-hoc* analysis of The Action in Diabetes and Vascular Disease: Preterax and Diamicron Modified Release Controlled Evaluation (ADVANCE) trial demonstrated that severe hypoglycemia was clearly associated with vascular events, as well as all-cause and cardiovascular mortality, with similar event rates observed in both the intensive and standard therapy groups. Importantly, severe hypoglycemia was also associated with non-cardiovascular outcomes, including respiratory, gastrointestinal, and dermatological events, supporting the interpretation that hypoglycemia may act as a marker of frailty and high-risk status rather than a cardiovascular-specific causal factor (6, 61). These findings are further supported by the Veterans Affairs Diabetes Trial (VADT), in which severe hypoglycemia was associated not only with major adverse cardiovascular events (MACE) and all-cause mortality, but also acted as a risk enhancer in patients with elevated baseline cardiovascular risk, where the association between hypoglycemia and cardiovascular events was even stronger. Notably, severe hypoglycemic episodes occurring within the preceding three months were associated with a higher subsequent rate of cardiovascular events (7). In the Outcome Reduction with Initial Glargine Intervention (ORIGIN) trial, the incidence of severe hypoglycemia was relatively low, however its occurrence was associated with an approximately 50% increase in relative risk across all major outcomes, including cardiovascular death, non-fatal myocardial infarction, stroke, all-cause mortality, cardiovascular mortality, and arrhythmic death, in patients with early type 2 diabetes or dysglycemia (63). Although the association between severe hypoglycemia in type 2 diabetes and cardiovascular events has been demonstrated in randomized clinical trials, a definitive causal relationship cannot be established.

A pivotal counterargument to a causal link between hypoglycemia and cardiovascular outcomes is provided by data from type 1 diabetes. In the Diabetes Control and Complications Trial (DCCT) in patients with type 1 diabetes, intensive glycemic control resulted in 40-70% reduction in microvascular complications and numerically lower incidence of cardiovascular events, despite a two to threefold increase in severe hypoglycemic episodes in intensive treatment arm (66). Long-term follow-up of the DCCT population in the Epidemiology of Diabetes Interventions and Complications (EDIC) study demonstrated a sustained reduction in cardiovascular events and preserved or improved cognitive outcomes in the intensive group (67, 68) Moreover, life expectancy was reported to be higher and comparable to that of the general population in intensively treated individuals (69).

Overall, while severe hypoglycemia is consistently associated with adverse cardiovascular and mortality outcomes, the mechanisms underlying these observations likely involving sympathetic activation, alterations in cardiac repolarization, and pro-inflammatory and pro-thrombotic pathways—appear to overlap substantially with pathways activated by hyperglycemia and advanced diabetes itself, supporting the concept of hypoglycemia primarily as a marker of increased cardiovascular vulnerability rather than an independent causal factor (6, 61, 63).

## 4 High-risk populations

High-risk populations with type 2 diabetes, such as individuals with long disease duration, coexisting cardiovascular disease, renal dysfunction, or advanced age, are particularly vulnerable to both hypoglycemia and cardiovascular events. In these patients, aggressive glucose-lowering strategies may paradoxically increase cardiovascular risk through episodes of severe hypoglycemia, which can trigger autonomic activation, arrhythmogenesis, endothelial dysfunction, and a pro-thrombotic state.

The magnitude of cardiovascular risk related to hypoglycemia is especially pronounced in individuals with pre-existing cardiovascular pathology or multiple comorbidities, suggesting a synergistic vulnerability where hypoglycemia acts as a precipitating biological stressor on compromised cardiovascular reserves (5–7, 63).

Population aging and frailty are becoming increasingly important in the context of cardiovascular outcomes. Aging is associated with a blunted counterregulatory response to hypoglycemia and likely contributes to more frequent silent hypoglycemic episodes, which cumulatively predispose to adverse cardiovascular consequences (70). In older adults from the Atherosclerosis Risk in Communities (ARIC) study population, severe hypoglycemia was associated with poorer cardiac function and worse cardiovascular outcomes (9). Additionally, in the older population, the presence of multiple comorbidities, frailty, and cognitive decline is further exacerbated by episodes of hypoglycemia (71).

Hypoglycemic episodes serve as a risk amplifier for cardiovascular events in patients with chronic kidney disease (CKD). The increased susceptibility to hypoglycemia in CKD is likely attributable to reduced renal clearance of antidiabetic medications and prolonged drug action (72, 73). Severe hypoglycemia is associated with microvascular complications and progressive renal dysfunction in patients with elevated baseline creatinine (74).

In individuals with existing cardiovascular disease or elevated baseline cardiovascular risk, the impact of hypoglycemia appears magnified. *Post-hoc* analyses from VADT and ACCORD demonstrated that patients with pre-existing atherosclerotic burden or prior cardiovascular events exhibited a stronger association between severe hypoglycemia and subsequent cardiovascular morbidity and mortality (75, 76). The presence of myocardial vulnerability, altered autonomic tone, and underlying endothelial dysfunction likely facilitates greater susceptibility to hypoglycemia-induced ischemia and arrhythmogenesis (77). Thus, in high-risk patients, hypoglycemia acts not merely as a metabolic disturbance but as a potent cardiovascular stressor, further emphasizing the necessity of individualized glycemic targets in this population.

## 5 Future perspectives

Current knowledge suggests a link between hypoglycemia and cardiovascular outcomes; however, it does not establish a temporal or causative relationship at this time (15). The concept of hypoglycemia as a risk factor for CV events has been increasingly challenged. The findings of the multinational The Cardiovascular and Renal Microvascular Outcome Study With Linagliptin (CARMELINA) trial indicated that hypoglycemia and CV outcomes are simply indicators of elevated risk, as the data demonstrated a bidirectional link between hypoglycemia and cardiovascular outcomes. However, another trial, the Cardiovascular Outcome Trial of Linagliptin vs Glimepiride in Type 2 Diabetes (CAROLINA), which primarily involved individuals with early-stage diabetes, revealed no correlation whatsoever, despite a three-to fivefold higher incidence of hypoglycemia in the glimepiride group (78). Conversely, in the Hypo-RESOLVE cohort, hypoglycemia was linked not only to acute cardiovascular disease and mortality but also to renal and retinal problems, as well as depression (79). Taken together, these findings support the concept that hypoglycemia may act primarily as a marker of increased vulnerability rather than a direct causal factor, although a contributory role in specific clinical contexts cannot be completely excluded.

Nonetheless, mechanistic understanding remain insufficient. Numerous proposed mechanisms elucidate how hypoglycemia induces cardiovascular events. It impacts cardiac function, increases the risk of arrhythmia and the likelihood of ischemia, hence potentially raising the danger of sudden cardiac death (9, 33, 80). Importantly, many of these mechanisms overlap with those attributed to hyperglycemia and advanced diabetes itself, complicating causal interpretation (81).

Particular focus should be directed towards patients at elevated risk, including those with numerous comorbidities, elderly, or pregnant women. The American Association of Clinical Endocrinology emphasizes the necessity of balancing glycemic management with the danger of hypoglycemia, particularly in at-risk populations (33, 82).

Future research should yield more comprehensive epidemiological evidence, novel or enhanced molecular insights regarding hypoglycemia and cardiovascular events, and ascertain whether hypoglycemia should be acknowledged as a modifiable cardiovascular risk factor.

## 6 Conclusion

Hypoglycemia represents a clinically significant and frequently underestimated indicator of increased cardiovascular risk. Extensive evidence associates hypoglycemia with autonomic dysfunction, proarrhythmic events, inflammation, and endothelial dysfunction, each of which constitutes a pathophysiological substrate for cardiovascular disease. While causality remains unconfirmed due to study heterogeneity and multiple confounding variables, current data indicate a strong association and imply that hypoglycemia may function predominantly as a marker of cardiovascular vulnerability, rather than an independent casual factor, although a contributory role in susceptible individuals cannot be entirely excluded. Additional research is required to elucidate causality, quantify risk amplification, and guide therapeutic strategies that optimize glycemic control while reducing hypoglycemia incidence.
